# A Rare Cause of Anterior Knee Pain: Radiological and Histopathological Analysis of a Prepatellar Subcutaneous Venous-Type Angioleiomyoma

**DOI:** 10.7759/cureus.78969

**Published:** 2025-02-13

**Authors:** Furkan Özdem, Hatice Türksoy, Ömer Ata, Rasime Pelin Kavak

**Affiliations:** 1 Department of Radiology, Health Sciences University Ankara Etlik City Hospital, Ankara, TUR; 2 Department of Pathology, Health Sciences University Ankara Etlik City Hospital, Ankara, TUR

**Keywords:** histopathological diagnosis, prepatellar knee pain, radiological imaging, subcutaneous lesions, venous-type angioleiomyoma

## Abstract

Angioleiomyoma is a rare benign tumor arising from the smooth muscle cells of vascular walls, with prepatellar localization being exceptionally uncommon. We present the case of a 30-year-old female patient with anterior knee pain and swelling, without a history of trauma or systemic disease. Physical examination revealed a mobile, well-circumscribed subcutaneous mass. Ultrasonography demonstrated a hypoechoic nodular lesion, and magnetic resonance imaging further characterized it as a well-defined soft tissue mass with iso-hyperintense T1-weighted and hyperintense proton density (PD) signals, exhibiting significant gadolinium enhancement. Surgical excision was performed, and histopathological analysis confirmed the diagnosis of a venous-type angioleiomyoma, with smooth muscle actin (SMA)-positive and desmin-positive spindle cells, lacking atypia or mitotic activity. Postoperatively, the patient’s symptoms resolved completely, with no recurrence. Given the rarity of prepatellar angioleiomyomas, their diagnosis can be challenging due to overlapping radiological features with other subcutaneous tumors, including hemangiomas and glomus tumors. This case underscores the importance of integrating clinical, radiological, and histopathological findings for accurate diagnosis and appropriate management. Early recognition and surgical excision are essential for symptom relief and favorable outcomes.

## Introduction

Angioleiomyoma, also known as vascular leiomyoma or angiomyoma, is a rare benign soft tissue tumor originating from smooth muscle cells and arising from the muscular layer of the vessel wall. The prevalence of the condition is highest among individuals between the ages of 40 and 60 years, with a higher incidence among the female population [1-5]. Angioleiomyoma can develop in various anatomical regions throughout the body. However, they most commonly present as painful solid masses in the subcutaneous tissue and dermis of the lower and upper extremities [1,2]. In the literature, numerous cases of angioleiomyomas localized specifically within the knee joint have been reported [5]. However, the prepatellar subcutaneous region is one of the rare anatomical locations where such lesions are observed [3,5]. In this study, we present a case of a subcutaneous venous-type angioleiomyoma, located in the prepatellar region with radiological and histopathological findings in a patient presenting with knee pain.

## Case presentation

A 30-year-old female patient presented with complaints of pain and swelling in the right knee. There was no trauma history or known chronic diseases. On physical examination, a painful and mobile subcutaneous mass was palpated in the anteroinferior region of the knee. The swelling was notable. There were no signs of inflammation, and laboratory findings were within normal limits. The patient’s knee range of motion was preserved, with no restriction in flexion or extension.

**Figure 1:**
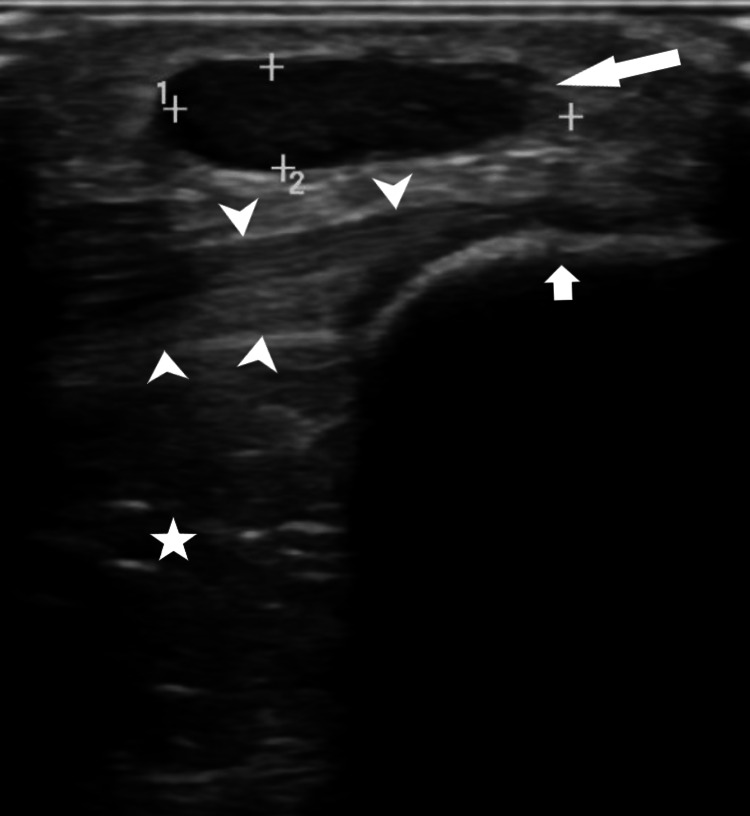
B-mode ultrasonographic examination demonstrates an oval-shaped, well-defined, hypoechoic nodular lesion with mild posterior acoustic enhancement located in the subcutaneous tissue of the prepatellar region, with its long axis parallel to the skin surface (long white arrow). The lesion is noted to be situated immediately anterior to the patella (short white arrow) and the proximal patellar tendon (white triangle arrowheads). No involvement or edema is observed in the infrapatellar fat pad (white star).

**Figure 2:**
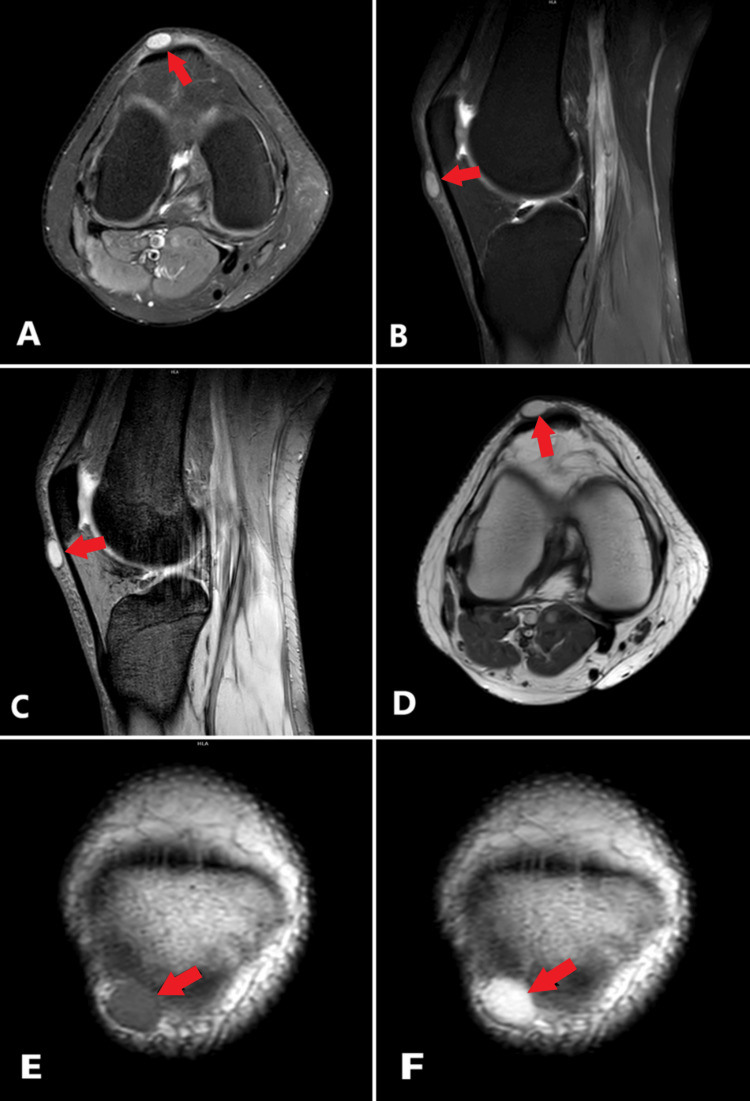
In the contrast-enhanced MRI examination of the right knee, the axial (A) and sagittal (B) proton density (PD)-weighted images show a well-defined hyperintense nodular lesion with capsular formation in the subcutaneous prepatellar area adjacent to the anteroinferior aspect of the patella and patellar tendon (red arrows). A similar hyperintense appearance was noted on multi-echo fast field echo (MFFE) T2-weighted images (C, red arrow). On coronal pre-contrast T1-weighted images (E), the lesion appeared homogenous and iso-hyperintense to muscle (red arrow). Post-contrast axial (D) and coronal T1-weighted images (F) demonstrated diffuse and homogeneous gadolinium enhancement of the lesion (red arrows).

On ultrasonography (USG), a well-defined, hypoechoic nodular lesion with mild posterior acoustic enhancement was observed in the subcutaneous tissue (Figure 1). Color Doppler USG revealed internal vascularization within the lesion. Subsequent MRI evaluation revealed the presence of a well-defined soft tissue lesion measuring approximately 9x6x6 mm. This lesion was located in the prepatellar region, extending within the subcutaneous fat planes in the plane of the knee joint. The lesion exhibited iso-hyperintense signal characteristics to muscle on T1-weighted images and hyperintense signal characteristics on proton density (PD) images. On post-contrast T1-weighted images, the tumor exhibited significant uniform gadolinium enhancement following contrast injection (Figure 2). The lesion showed no significant invasion or relationship with the tendons or neurovascular components in the subcutaneous tissue. There was no evidence of effusion in the knee joint. Adjacent soft tissues appeared normal. The menisci and tendons were unremarkable.

**Figure 3:**
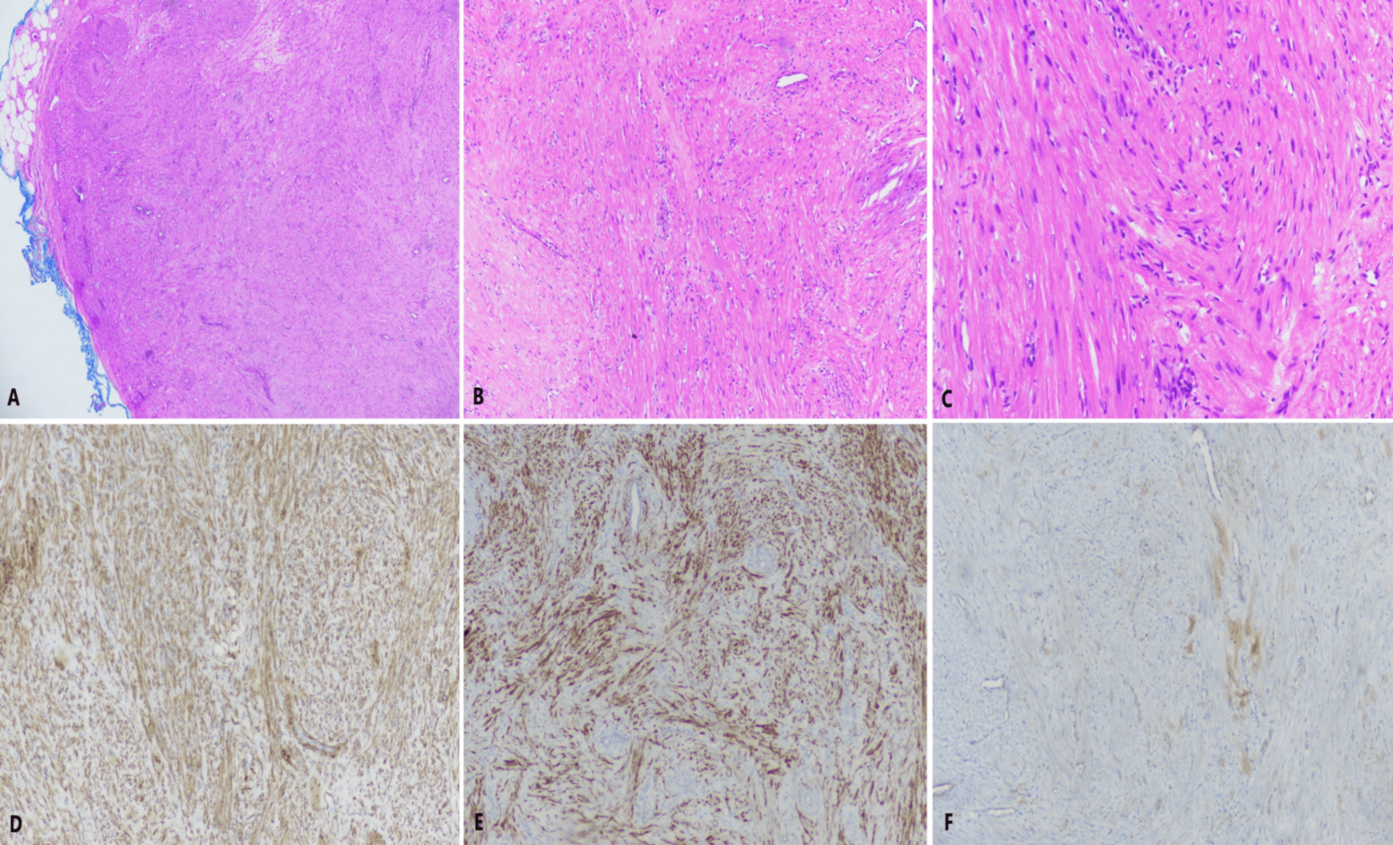
Histochemical and immunohistochemical analyses (A) Encapsulated proliferation of eosinophilic spindle-shaped smooth muscle cells, accompanied by thick-walled and occasionally slit-like venous vessels interspersed among smooth muscle fibers (H&E, 40x). (B) Fascicles of spindle-shaped smooth muscle cells without atypia or mitotic activity (H&E, 100x). (C) Spindle-shaped cells with cigar-shaped nuclei and abundant eosinophilic cytoplasm (H&E, 200x). (D) Diffuse positive staining of tumor cells with smooth muscle actin (SMA, 100x). (E) Widespread positive staining of tumor cells with desmin (100x). (F) Negative staining of tumor cells with S100 (100x).

On the basis of the ultrasonographic and MRI findings, surgical excision was considered an appropriate intervention, and an excisional biopsy of the lesion was performed. Macroscopic evaluation revealed that the excised tissue specimen had a well-circumscribed, smooth surface, was encapsulated, and exhibited a pink coloration. Histochemical and immunohistochemical analyses revealed that the lesion was smooth muscle actin (SMA)-positive (+), desmin-positive (+), and S100-negative (-). The smooth muscle cells exhibited no cellular atypia, and mitotic figures were few. Based on these findings, the lesion was diagnosed as a "venous-type angioleiomyoma" (Figure 3). Postoperatively, it was reported that the patient's complaints of swelling and pain in the right knee had completely resolved.

## Discussion

Leiomyomas are benign neoplasms originating from smooth muscle cells and constitute approximately 4.4-5% of all benign soft tissue tumors [2-19]. Based on the type of smooth muscle cells from which they originate, they are classified as angioleiomyoma, piloleiomyoma, and genital leiomyoma [16].

Angioleiomyoma, or vascular leiomyoma, is a benign solitary soft tissue tumor originating from the tunica media of blood vessels [5]. It is more commonly observed in middle-aged women [3,5]. The exact etiology is unknown; however, trauma, infections, hormonal changes, vascular anomalies, and genetic factors are considered potential contributors to the pathogenesis [19]. Some studies have indicated that these lesions can be observed in various parts of the body, such as the cranium, atrium, uterus, urethra, nasal septum, soft palate, eyelid, hands, feet, etc. [7,8]. However, these tumors are most commonly seen in the lower extremities within the subcutaneous soft tissue [5-7]. The knee joint, particularly the prepatellar region, is an exceptionally rare site for these lesions. The existing research on the subject indicates that cases originating in the prepatellar region and confirmed histologically as venous type are rare. Considering that the prepatellar region primarily comprises adipose tissue, the origin of the lesion is thought to be associated with the venous capillaries within the fat tissue.

Angioleiomyomas are generally characterized by an asymptomatic presentation. However, as observed in the present case, these lesions may manifest as painful, mobile, and less than 2 cm nodular formations in the subcutaneous soft tissue [7,8]. The differential diagnosis encompasses a wide range of conditions associated with knee pain, including meniscus or tendon injuries, degenerative processes, bursitis, arthritis, and a variety of nodular lesions. These include hemangiomas, angiolipomas, lipomas, inclusion cysts, ganglion cysts, glomus tumors, fibromas, schwannomas, synovial sarcomas, loose bodies, pigmented villonodular synovitis (PVNS), leiomyosarcomas, and giant cell tumor of the tendon sheath [3-8].

The radiological diagnosis of angioleiomyomas is often challenging since they do not exhibit distinctive imaging characteristics. Patients with knee pain and a palpable mass are usually evaluated with plain X-rays and USG followed by MRI for further assessment. Plain X-rays may show calcifications in the lesion but are not often used as a primary diagnostic method [7]. Aydın et al. noted that plain orthogonal radiographs revealed soft tissue swelling on the lateral aspect of the patella [3].

As demonstrated in this case, USG is a diagnostic tool that can reveal the shape, margins, and characteristics of subcutaneous lesions (Figure 1). Cantisani et al. described angioleiomyomas on ultrasound as subcutaneous, oval, well-defined, slightly hypoechoic lesions with diffuse and peripheral vascularization [6]. Similarly, in our case, the lesion demonstrated these characteristic features, including well-defined margins, a slightly hypoechoic appearance, and internal vascularization on color Doppler USG. In a study by Ramesh et al., the use of color Doppler imaging in the examination of angioleiomyomas revealed a high degree of resistance in the arteries located within the tumors. This finding suggests the potential presence of muscular arteries within these tumors [9].

MRI is a preferred method for detailed assessment of lesions and clarification of their relationship with adjacent soft tissues. MRI typically reveals well-defined lesions that manifest as round or oval-shaped lesions. These lesions exhibit iso-hyperintense characteristics on T1-weighted images relative to muscle tissue and are characterized by hyperintense signals on T2-weighted images [4-13]. Hwang et al. suggested that the peripheral hypointense area on T2-weighted images shows the fibrous capsule in vascular leiomyomas [11]. Approximately 94% of lesions demonstrate diffuse and homogeneous contrast enhancement following gadolinium administration [2]. In a study, the lesion was described as exhibiting only peripheral enhancement [10]. Yoo et al. reported fewer vascular structures on microscopic examination in patients with only peripheral enhancement [12].

The first comprehensive study on angioleiomyomas was published in the literature by Stout in 1937 [18]. Angiomyoleiomyomas are classified into three histological types by Moromoto [14] and Hachisuga et al. [15]: solid (capillary), venous, and cavernous. The most common type is solid (capillary) (approximately 66% of cases). The cavernous type is the rarest (representing 11% of all cases), and the venous type is the second most common (23% of cases) [8,14,15]. Venous and cavernous types are typically painless, whereas solid tumors are associated with pain [14]. The solid type has tightly packed smooth muscle fibers and many small blood vessels, while the cavernous type contains large, dilated blood vessels and less smooth muscle. As in our case, venous-type angiomyoleiomyomas are composed of thick vascular walls with intravascular smooth muscle [14,15]. Matsuyama et al. analyzed 122 cases of angiomyoleiomyomas (74 solid, 37 venous, and 11 cavernous types) and showed that all tumor cells were positive for SMA, with 51.4% of venous types showing diffuse positivity for desmin [17]. In a study involving 142 cases of angioleiomyomas, no cellular atypia or malignant transformation was observed [20].

## Conclusions

Prepatellar subcutaneous venous-type angioleiomyomas are a rare cause of anterior knee pain. An accurate diagnosis necessitates meticulous radiological and histopathological evaluation, attributable to the absence of specificity in clinical manifestations and imaging findings. Surgical excision provided complete symptom relief in this case, emphasizing the importance of timely diagnosis and treatment. Further research is needed to clarify the etiology and clinical characteristics of these rare tumors.
